# Mortality following hip fracture surgery in patients with dementia: a Swedish multiple national register study

**DOI:** 10.1007/s41999-025-01163-6

**Published:** 2025-02-23

**Authors:** Michael Axenhus, Sara J. Hägg, Maria Eriksdotter, Margareta Hedström, Dorota Religa

**Affiliations:** 1 Department of Orthopaedic Surgery, Orthopaedic Clinic, Danderyd Hospital, Danderyd University Hospital, Entrévägen 2, 182 68 Danderyd, Sweden; 2 Department of Clinical Sciences, Danderyd Hospital, Karolinska Institutet, Stockholm, Sweden; 3 Department of Medical Epidemiology and Biostatistics, Karolinska Institutet, Stockholm, Sweden; 4 Department of Neurobiology, Care Sciences and Society, Division of Clinical Geriatrics, Karolinska Institutet, Stockholm, Sweden; 5 Inflammation and Aging Theme, Karolinska University Hospital, Stockholm, Sweden; 6 Department of Clinical Science, Intervention and Technology (CLINTEC), Karolinska Institutet, Stockholm, Sweden; 7 Trauma and Reparative Medicine Theme (TRM), Karolinska University Hospital, Stockholm, Sweden

**Keywords:** Hip fracture, Dementia, Mortality, Risk factors, Sweden

## Abstract

**Purpose:**

Hip fractures in older adults are a significant risk factor for morbidity and mortality, with the presence of dementia further complicating outcomes. The purpose of this study was to investigate the association between hip fractures and mortality of patients with dementia and dementia types in comparison to those without dementia following hip fracture surgery.

**Methods:**

Utilizing data from the Swedish Hip Fracture Register (SHR), Swedish Registry for Cognitive/Dementia Disorders (SveDem), National Patient Register (NPR), and National Prescribed Drug Register (PDR), we conducted a retrospective analysis of 111,353 patients aged 65 and older who underwent hip fracture surgery between 2010 and 2018. Patients were categorized into two cohorts: those with and without a known diagnosis of dementia prior to the hip fracture. Multivariable Cox regression analyses were used to evaluate mortality risk factors.

**Results:**

Of the study sample, 22% had dementia. Dementia patients exhibited higher mortality rates at 30 days with 13% vs. 6%, (*p* < 0.001), 4 months with 27% vs. 12%, (*p* < 0.001) and at 1 year with 39% vs. 20%, post-fracture (*p* < 0.001). Higher ASA grades, poor baseline walking ability, and long-term care residency were also associated with increased mortality. Parkinson’s disease dementia was associated with a higher mortality compared to other dementias during the first 4 months post-operatively.

**Conclusion:**

Our findings revealed an association between dementia diagnosis and higher risk of mortality following hip fracture surgery. These findings underscore the need for specialized post-operative care. Involving specific post-operative geriatric competence, such as orthogeriatric or orthogeriatric models of care could potentially improve outcomes. Further research is needed to explore the impact of dementia severity and subtype on mortality.

**Supplementary Information:**

The online version contains supplementary material available at 10.1007/s41999-025-01163-6.

## Key summary points

**Aim:**

This study aimed to investigate whether dementia is associated with increased mortality following hip fractures and how different dementia subtypes affect mortality outcomes.

**Findings:**

The study found that patients with dementia had significantly higher mortality rates at 30 days, 4 months, and 1-year post-hip fracture compared to those without a diagnosis of dementia. Subtypes such as Parkinson’s disease dementia and dementia with Lewy bodies posed particularly high risks.

**Message:**

Dementia significantly increases mortality after hip fractures, especially in certain subtypes, highlighting the need for tailored post-operative care in these patients.

**Supplementary Information:**

The online version contains supplementary material available at 10.1007/s41999-025-01163-6.

## Introduction

Hip fractures are a risk factor for morbidity and mortality among the older adult population [1, 2]. The life risk for hip fracture is about 11% for men and double for women [3, 4]. The mortality due to complications and associated diseases following hip fracture surgery is significant, prompting research on this vulnerable patient population [5].

Dementia has previously been shown to increase the risk of hip fractures [6]. The incidence of dementia and cognitive impairment among patients who suffer hip fractures in Sweden have been estimated to around 12–20% [7, 8]. Dementia has been identified as an independent mortality risk for hip fracture patients [6, 7, 9, 10]. Furthermore, dementia is one of the most important risk factors for delirium which increases the risk for post-operative complications and mortality [11]. Hip fracture patients may benefit from post-operative care in geriatric wards or environment with geriatric competences [12].

The causes for mortality following a hip fracture in patients with dementia is not well understood. The current literature is limited by small sample size studies [9, 10, 13, 14] and inadequate adjustments for important confounding factors such as prior residential status and walking ability [6, 7, 15]. There is also a knowledge gap regarding the impact of various subtypes of dementia on the mortality of hip fracture patients. It is well known that different types of dementia impact patients differently.

The purpose of this study is to investigate the association between hip fractures and mortality of patients with dementia and dementia types in comparison to those without dementia following hip fracture surgery.

## Methods

### Study design

This is a prospective register study utilizing data from RIKSHÖFT, the Swedish National Registry for Hip Fractures (SHR). The data obtained were collected between January 1st, 2010, and December 31st, 2018. The SHR data were linked with the Swedish Registry for Cognitive/Dementia Disorders (SveDem), the National Patient Register (NPR), and the National Prescribed Drug Register (PDR) to identify patients with dementia. The Swedish National Cause of Death Register provided the dates of death. The linkage was conducted using patients’ unique personal identity numbers which is assigned to every resident in Sweden. The STROBE recommendations and the Helsinki Declaration were followed [16, 17].

### Inclusion and exclusion

The following variables were obtained from SHR where available: age, sex, ASA grade, fracture date, fracture type, residential status, cognitive status, discharge destination, baseline walking ability, and walking ability four months after the fracture date. Information about dementia diagnosis to the registry and residential status was provided either by the patient, next of kin, or nursing staff with prior knowledge of the patient.

We included all patients with a surgically treated hip fracture over the age of 65. We excluded patients under the age of 65 years, those with non-operatively treated hip fractures, or those with pathological fractures. For the survival analysis, patients with missing values for ASA grade, residential status, or baseline walking ability were excluded. In the SHR, individuals with missing cognitive state information were excluded.

### Registers and categorization of dementia

Diagnostic examinations for dementia in Sweden are conducted by primary care physicians (basic work-up) in the first step (50%of cases stay on that level) and later in memory clinics if needed by geriatricians, neurologists, or old age psychiatrists. The diagnostic process takes usually 3 months [18]. The SHR has collected information since 1988 and contains data on more than 300 000 hip fractures [19]. It has a coverage above 80% for the hip fracture population of Sweden [20]. The cognitive state of a patient was registered in SHR as either “fully oriented”, “not fully oriented” or “known dementia”. Patients categorized with “known dementia” were considered to have dementia. Information about the patient’s cognitive status is recorded in the SHR by the relevant healthcare professional as part of the care process during hip fracture treatment. SveDem is a national quality registry in Sweden which register persons at the time of the dementia diagnosis and evaluate and follow-up annually the quality of diagnostics, treatment, and care for patients with various dementia disorders [18]. Patients registered in SveDem with a dementia diagnosis prior to the hip fracture were considered to have dementia. Diagnosing physicians, often in primary care or memory clinics, enter dementia diagnoses into SveDem. This includes specific dementia subtypes, diagnostic methods, and details about patient care and treatment. Memory clinics are available in 20 of Sweden’s 21 regions, though accessibility varies, with some regions referring patients to neighboring areas. National diagnostic guidelines from the Swedish National Board of Health and Welfare guide care, and in 2024, 92% of dementia patients underwent a baseline diagnostic evaluation as per these guidelines.

The NPR and the National Cause of Death Register are validated administrative registers with close to full national coverage in Sweden [21]. The NPR register all diagnoses made in Sweden, in inpatient and outpatient specialist clinics but not primary care [22]. Dementia diagnoses are documented in NPR by healthcare professionals during inpatient or outpatient specialist visits using International Classification of Diseases (ICD) codes ICD codes. Patients with one of the following ICD codes: F0 [0–4], F051, G3 [0–1], or A810 registered between 2007–2018 prior to the hip fracture were considered to have dementia. Subgroups of dementia was classified as; late Alzheimer disease (AD), early AD, vascular dementia (VascD), mix of AD and VascD, frontotemporal dementia, dementia with Lewy bodies, Parkinson disease dementia, no specified dementia, and other dementias. Different dementia types are reported according to the classification noted in SveDem.

The PDR is a Swedish national registry which contains information on all drugs dispensed on prescription at pharmacies since 2005, and it also include most drugs dispensed in long-term care facilities but not hospital [23]. The registry uses the Anatomical Therapeutic Chemical (ATC) classification system, with the N06D code identifying anti-dementia medications. Dementia-related medications, such as cholinesterase inhibitors, are automatically registered when dispensed, providing a supplementary source of diagnostic data. To identify patients with dementia, we included patients who had received anti-dementia drugs between 2007 and 2018, prior to their hip fracture date.

We combined all cases with a diagnosis of dementia identified in either SHR, SveDem, NPR, and PDR into a single group called “Dementia”. The diagnostic sensitivity of dementia was 77% in SHR compared to dementia diagnoses identified in PDR, NPR, or SveDem in a pooled group. The largest overlap of dementia diagnosis was between NPR and SHR (Supplemental Fig. S1).

### Statistical analysis

Categorical variables are shown as percentages, while continuous variables are displayed as the mean and interquartile range (IQR). The Wilcoxon rank sum test was used to analyze differences between continuous variables, and Pearson’s Chi-squared test or Fisher’s exact test was used for categorical variables. Missing data were addressed using complete case analysis. Statistical significance was determined by a two-sided *p* value of less than 0.05 and a 95% confidence interval (CI).

The hazard ratio (HR) for mortality was calculated using multivariable Cox regression analysis. Univariate analyses were conducted for variables including dementia type, ASA grade, sex, age, residential status, baseline walking ability, and fracture type. Variables that were statistically significant or did not amplify the impact of dementia were included in the multivariable analysis. All statistical analyses were performed using SPSS version 29.0 [24].

## Results

### Study material

The study sample includes 87,363 patients without dementia (78%) and 23,990 with dementia (22%). Patients with dementia were older, with a median age of 86 years vs 83 years for those without dementia (*p* < 0.001). Higher ASA grades were more common in dementia patients, with 62% having a grade of 3 compared to 48% in the non-dementia group, and 9.6% having a grade of 4 compared to 7.1% (*p* < 0.001). Mortality rates were higher for dementia patients: 30-day mortality was 13% compared to 6% for non-dementia patients; 4-month mortality was 27% vs 12%; and 1-year mortality was 39% compared to 20% (*p* < 0.001) (Supplementary Table S1). There was a total of 5,944 patients with dementia where different dementia disorders were specified, of which those with unspecified dementia and late AD being the most common with *n* = 1575 and *n* = 1533 cases, respectively. Patients with early AD and frontotemporal dementia had the lowest number of cases with *n* = 74 and *n* = 46. Follow-up time from initial surgery was longest for those with Parkinson disease dementia with an average follow-up of 821 days (Supplementary Table S2).

### Mortality

**Fig. 1:**
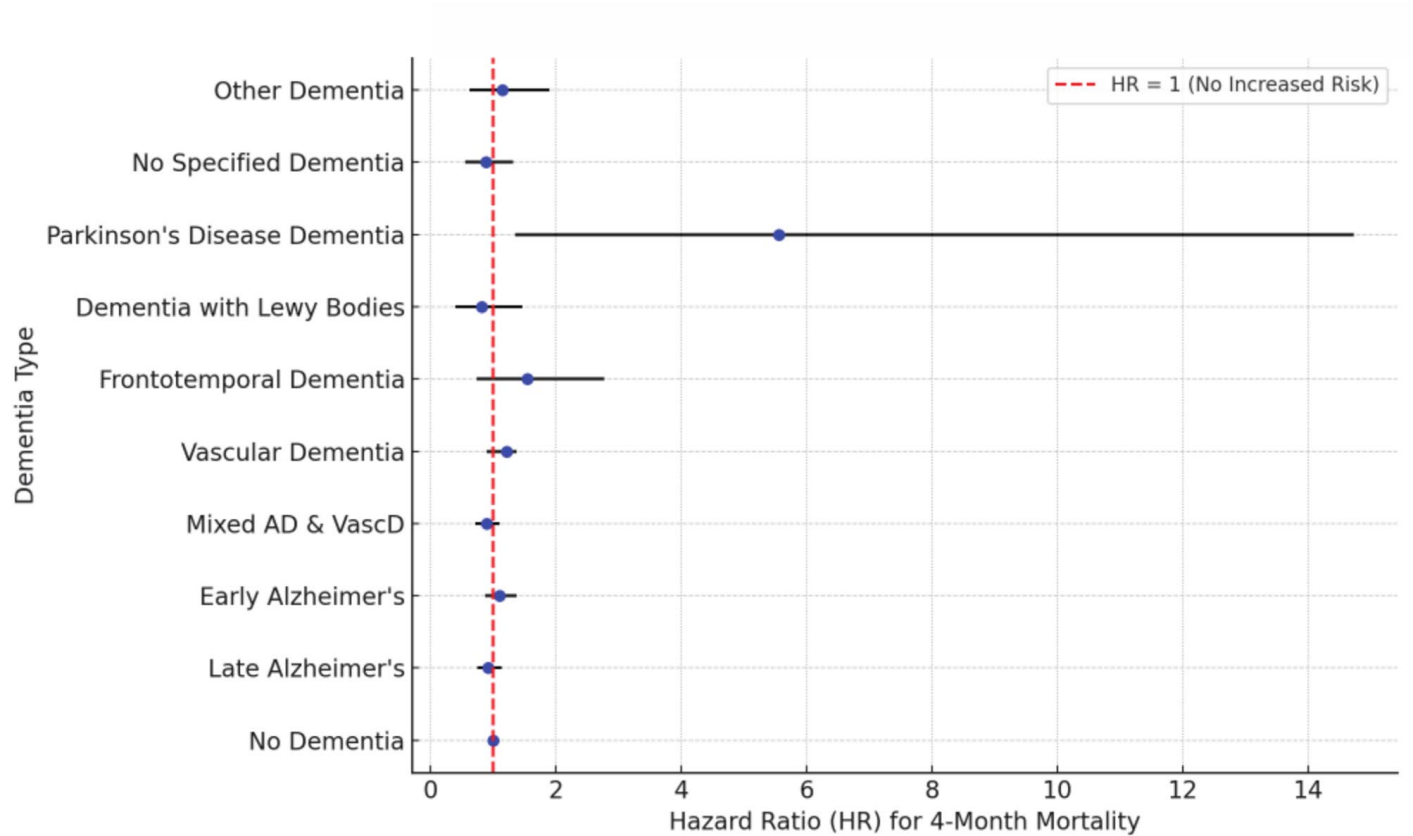
Hazard ratios of 4-month mortality per dementia type

At 4 months, patients with dementia had a 26% higher risk of mortality compared to patients without (no known) dementia. Among patients with different dementia types, patients with Parkinson disease dementia showed a significantly higher risk of mortality (HR: 5.56) compared to patients without dementia, while patients with other types of dementia did not show higher risk. Grade of orientation was associated with increased mortality. Several covariates were significantly associated with increased 4-month mortality following hip fracture surgery. High ASA grades, advanced age, male sex, poorer baseline walking ability, and residence in long-term care facilities showed associations with elevated mortality risk (Fig. 1) (Table 1). At 1 year, there was no significant difference in mortality between patients with dementia and patient without (Supplemental Table S3) (Supplemental Fig. S2).

**Table 1:** Multivariable Cox regression analysis for risk factors of 4-month mortality following a hip fracture

	HR	95% CI	*p* value
No dementia	REF	–	
Dementia	1.26	1.22–1.31	< 0.001
Late AD	0.92	0.77–1.11	
Early AD	1.1	0.89–1.35	
Mix of AD and VascD	0.89	0.74–1.08	
VascD	1.21	0.92–1.35	
Frontotemporal dementia	1.54	0.76–2.75	
Dementia with Lewy bodies	0.82	0.42–1.44	
Parkinson disease dementia	5.56	1.37–14.71	
No specified dementia	0.88	0.58–1.29	
Other dementia	1.14	0.65–1.87	
Cognitive state			< 0.001
Fully oriented	–		
Not fully oriented	1.38	1.34–1.43	
Known dementia	1.63	1.58–1.67	
Sex (women)	0.57	0.55–0.59	< 0.001
ASA grade			< 0.001
1	–	–	
2	1.54	1.33 to1.79	
3	2.77	2.40–3.20	
4	5.57	4.81–6.46	
5	9.93	7.45–13.2	
Age	1.06	1.06–1.06	< 0.001
Baseline walking ability			< 0.001
Alone outdoors	–	–	
Only with company outdoors	1.63	1.55–1.72	
Alone indoors	1.72	1.65–1.79	
Only with company indoors	1.81	1.71–1.91	
Can't walk	1.88	1.75–2.03	
Fracture type			< 0.001
Nondisplaced cervical (Garden 1–2)	–	–	
Displaced cervical (Garden 3–4)	1.17	1.11–1.23	
Basocervical	1.17	1.07–1.29	
Intertrochanteric (two-part)	1.14	1.08–1.21	
Intertrochanteric (multiple parts)	1.21	1.15–1.28	
Subtrochanteric	1.22	1.14–1.31	
Residential status			< 0.001
Single person household	–	–	
Multiple person household	0.95	0.91–0.99	
Long-term care facility	1.45	1.40–1.51	

HR, Hazard Ratio; CI, Confidence Interval; ASA, American Society of Anesthesiology

**Fig. 2:**
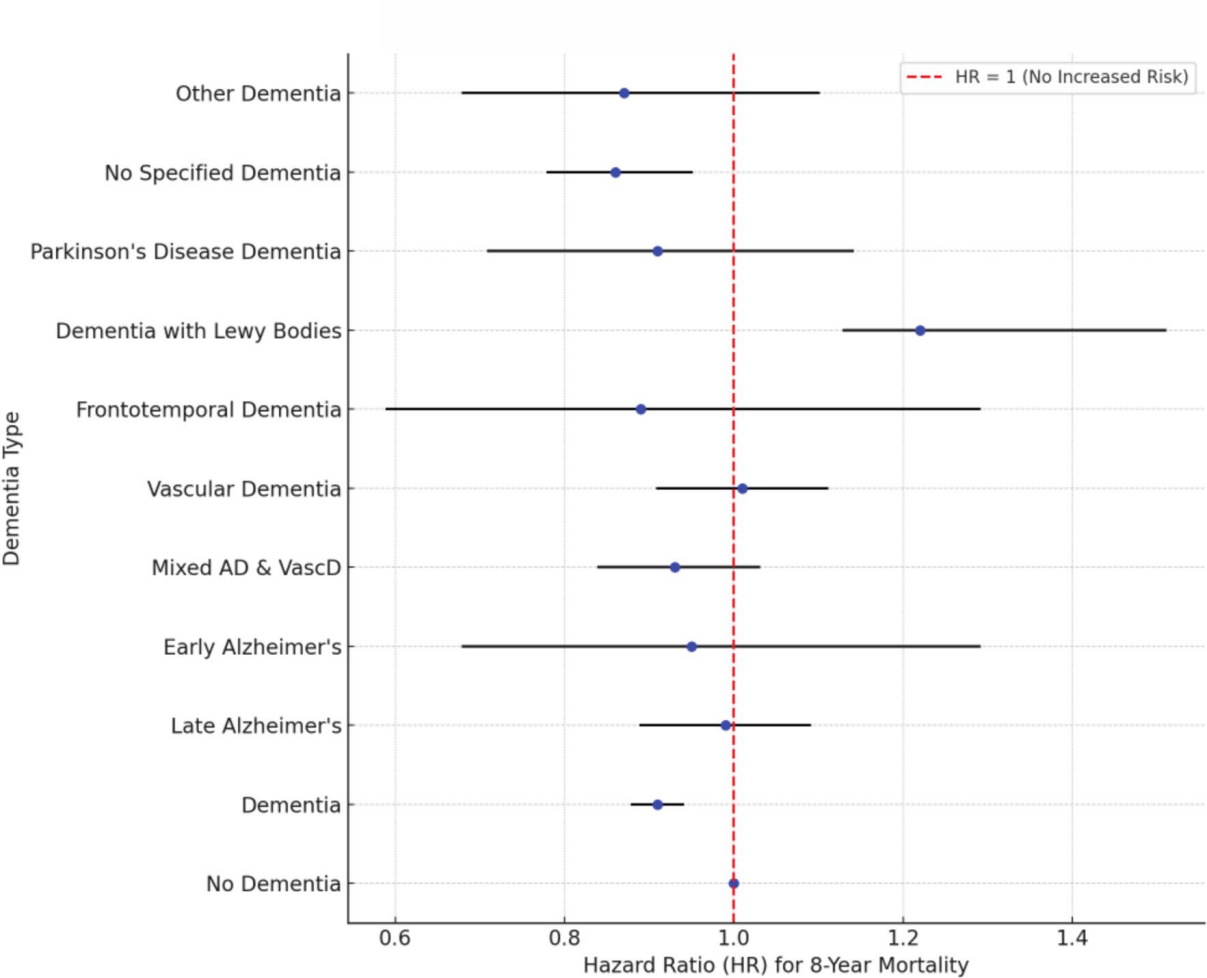
Hazard ratios of 8-year mortality per dementia type

Up to 8 years post-surgery, data from the National Cause of Death Register showed that patients with dementia had a significantly reduced risk of mortality (Fig. 2) (Table 2).

**Table 2:** Dementia mortality up to 8 years following surgically treated hip fractures

	HR	95% CI	
No dementia	–	–	
Dementia	0.91	0.88–0.94	< 0.001
Late AD	0.99	0.89–1.09	
Early AD	0.95	0.68–1.29	
Mix of AD and VascD	0.93	0.84–1.03	
VascD	1.01	0.91–1.11	
Frontotemporal dementia	0.89	0.59–1.29	
Dementia with Lewy bodies	1.22	1.13–1.51	
Parkinson disease dementia	0.91	0.71–1.14	
No specified dementia	0.86	0.78–0.95	
Other dementia	0.87	0.68–1.10	
Sex (women)	0.80	0.79–0.82	< 0.001
ASA grade			< 0.001
1	–	–	
2	1.42	1.21 to1.67	
3	2.53	2.16–2.96	
4	5.12	4.36–6.01	
5	8.72	6.24–12.0	

HR, hazard ratio; CI, confidence interval**;** ASA, American Society of Anesthesiology

**Fig. 3:**
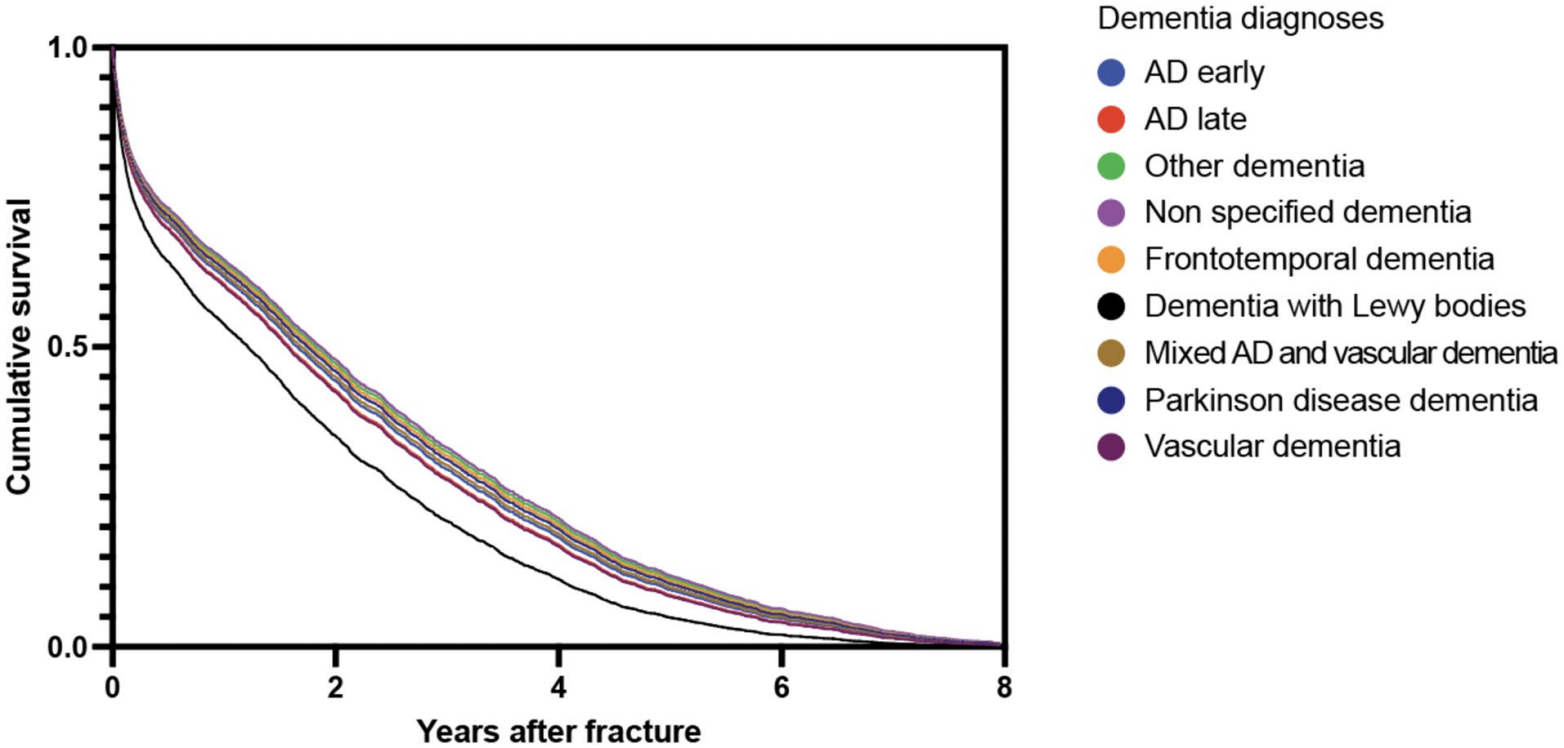
Survival following surgically treated hip fractures among patients with dementia. Dementia types are adjusted for ASA grade, sex, age, residential status, baseline walking ability, and fracture type. Lines indicate patients with specific dementia types

In contrast, patients with dementia with Lewy bodies had an increased risk (HR: 1.22) (Table 2) (Fig. 3). Patients with ASA grade > 1 also had significantly increased mortality risk.

## Discussion

This study highlights the significant impact of dementia on mortality rates following hip fractures in older adults with or without dementia. Our study found that patients with dementia have a notably higher risk of mortality at 30-day, 4-month, and 1-year post-fracture compared to those without dementia, although this was the case during long-term follow-up with patients with dementia displaying slightly decreased mortality at 8-year post-surgery. This aligns with previous studies that have identified dementia as a critical risk factor for adverse outcomes in hip fracture patients [25, 26].

The increased mortality risk in dementia patients can be attributed to several factors. Dementia is associated with higher ASA grades, indicating more severe comorbid conditions, which likely contribute to poorer outcomes. In addition, dementia patients with hip fractures are more frequently residents of long-term care facilities and have compromised baseline walking abilities, both of which are associated with increased mortality [7, 27–29].

Our analysis of dementia subtypes reveals that not all dementias confer the same risk. Dementia with Lewy bodies significantly increase mortality risk during the long-term follow-up, while Parkinson disease dementia had a high mortality in the short-term follow-up, underscoring the need for targeted interventions in these subgroups. Interestingly, late Alzheimer’s disease and other mixed dementias did not significantly increase risk compared to the reference group, suggesting variability in how different dementia pathologies interact with hip fracture outcomes. This is in line with other studies that have highlighted variance in dementia mortality depending on subgroups [30, 31]. Sex differences also emerged, with women showing a lower risk of mortality, which may be related to differences in disease presentation and comorbidities between men and women. Age and higher ASA grades were consistent predictors of increased mortality, reinforcing the importance of these factors in clinical risk assessment. The cognitive level probably also plays a role which was not included here [32].

A previous study using data from the NPR found a 30-day mortality risk of 67% for patients with dementia, corresponding to an absolute risk difference of 4.2% [7]. We observed a 30-day mortality risk increase of 22% for dementia patients, resulting in an absolute risk difference of 1.4%. The variation in findings underscores the critical importance of accounting for an individual’s level of dependence when assessing the impact of dementia on mortality. Factors such as living situation in a long-term care facility and baseline walking ability are crucial for accurately estimating risk. By adjusting for these variables, our study aimed to minimize the risk of overestimating the impact of dementia on mortality. It is likely that this discrepancy is due to our use of multiple data sources to enhance the sensitivity of dementia identification, as well as differences in the adjustments made for confounding factors such as residential status.

SHR accurately reflects the hip fracture population in Sweden and has a coverage of 80% [20]. Sweden has publicly funded universal health care system, which is of high quality internationally and all Swedish citizens receive similar access and quality of health care. Though local guidelines differ in Sweden, there are central guidelines which all health care providers adhere to. The results in this study have, therefore, high generalizability to the Swedish hip fracture population. Outside of Sweden, the result can best be generalized to other western countries with similar hip fracture treatment and access. It is worth to note that living in long-term care facility might have a larger impact on mortality in Sweden than other western countries. This is due to individuals in long-term care facility homes in Sweden are generally of poor health and more than half of those admitted dies within 2 years [33].

This study underscores the necessity of specialized post-operative care for dementia patients, such as admission to geriatric wards, which has been shown to improve outcomes [28, 34]. The high prevalence of dementia among hip fracture patients and its impact on mortality emphasize the need for comprehensive care strategies that address both the cognitive and physical health of these patients. This is particularly important as a recent study have found that patients with dementia have reduced walking ability following hip fractures [35].

Geriatric or orthogeriatric care models can significantly improve outcomes for patients with hip fractures. It has been shown that patients treated in geriatric units have significantly lower mortality rates compared to those treated in orthopedic units [28, 34, 36]. Dementia patients benefited the most from geriatric care, with an average of 3.2 days of post-operative delirium in the geriatric care group versus 12.8 days in the orthopedic care group. A study by Pajulammi et al. have also showed that patients with known cognitive disorder or dementia can benefit from a comprehensive geriatric assessment (CGA) during acute hip fracture care in regards to short-term mortality [37]. Taken together, these studies indicate that a possible mitigator of increased mortality following hip fractures in the older adults might be the utilization of post-operative geriatric care, in particularly orthogeriatrics and the use of CGA, among those with dementia.

It remains unclear how symptom severity affects mortality outcomes for dementia patients with hip fractures. Varying degrees of dementia symptomology might influence mortality differently. This has been suggested by a study of Tarazona-Santabalbina et al. [32], where patients, following a hip fracture surgery, with mild and moderate dementia had no increased mortality risk and only for patients with severe dementia had an associated increase in mortality. However, a similar study by Schaller et al. found mild and moderate dementia to be a major risk factor following a hip fracture [29]. Nevertheless, dementia patients are a vulnerable patient population following orthopedic injuries, and further research is warranted.

Even though SveDem has national coverage, it should be noted that participation in SveDem is voluntary, and no mandatory comprehensive register of dementia exists in Sweden. Our use of multiple register to identify dementia patients is a comprehensive approach but it does not guarantee complete coverage. In addition, all persons with dementia are not identified by the health care system. For example, in a meta-analysis by Seitz et al., 20% of hip fracture patients had dementia which is in line with our study where 22% of the sample had dementia. However, the included studies had a lower mean age than compared to our population. Furthermore, patients with dementia receive less hospital-based rehabilitation after a hip fracture compared to their counterparts [38] which is not controlled for in this study. It is also important to note that a significant proportion of patients were classified as non-specified dementia. This may have impacted the reliability of the results for specific dementia subtypes by potentially underestimating or misclassifying certain cases. Lastly, our study did not adjust for dementia severity or malnutrition, which are both factors that influence mortality outcomes in dementia patients.

## Conclusion

In conclusion, this study highlights the significant impact of dementia on mortality following hip fractures in older adults. Patients with dementia face higher mortality rates at 30-day, 4-month, and 1-year post-fracture compared to those without dementia. Factors such as higher ASA grades, poorer baseline walking abilities, and living in long-term care facilities contribute to this increased risk.

Our findings reveal an association between dementia diagnosis and higher risk of mortality following hip fracture surgery. This underscores the need for specialized post-operative care, particularly in geriatric wards, to improve outcomes for dementia patients.

## Supplementary Information

Below is the link to the electronic supplementary material.

Supplementary file1 (DOCX 1008 kb)

## Data Availability

The data used in this study contain sensitive individual-level information and is protected by the Swedish Personal Data Act. Consequently, sharing the data requires ethical approval and consent from the principal investigator.
