# Supplementary material for: Mortality following hip fracture surgery in patients with dementia: a Swedish multiple national register study
Source: Eur Geriatr Med. 2025 Feb 23;16(2):541–9. doi: 10.1007/s41999-025-01163-6 (PMC12014816; doi:10.1007/s41999-025-01163-6)
Supplement: Supplementary file 1 — Supplementary file1 (DOCX 1008 kb) [file 41999_2025_1163_MOESM1_ESM.docx]

# Supplementary material

**Supplementary tTble 1:** The demographic and clinical characteristics of hip fracture patients in the survival analysis. Percentage is given as the percentage per variable.

| **Variable** | **No dementia**, N = 87363, percentage = (78)^‡^ | **Dementia**, N = 23990  percentage = (22)^‡^ | **p-value**^§^ |
| --- | --- | --- | --- |
| **Age, median (range)** | 83 (76, 89) | 86 (81, 90) | <.001 |
| **ASA**^\|\|^ **grade** |  |  | <.001 |
| **1** | 5,312 (6.1) | 261 (1.1) |  |
| **2** | 33,771 (39) | 6,482 (27) |  |
| **3** | 41,988 (48) | 14,897 (62) |  |
| **4** | 6,219 (7.1) | 2,313 (9.6) |  |
| **5** | 73 (<0.1) | 37 (0.2) |  |
| **Sex** |  |  | <.001 |
| **Men** | 28,206 (32) | 7,343 (31) |  |
| **Women** | 59,157 (68) | 16,647 (69) |  |
| **Cognitive state** |  |  | <.001 |
| **Fully oriented** | 52,865 (84) | 1,165 (6.0) |  |
| **Not fully oriented** | 10,214 (16) | 1,899 (9.8) |  |
| **Known dementia** | 0 (0) | 16,304 (84) |  |
| **(Missing)** | 24,284 | 4,622 |  |
| **Fracture type** |  |  | <.001 |
| **Nondisplaced cervical (Garden 1–2)** | 11,148 (13) | 3,159 (13) |  |
| **Displaced cervical (Garden 3–4)** | 33,335 (38) | 9,295 (39) |  |
| **Basocervical** | 2,823 (3.2) | 790 (3.3) |  |
| **Intertrochanteric (two-part)** | 16,659 (19) | 4,522 (19) |  |
| **Intertrochanteric (multiple parts)** | 16,259 (19) | 4,545 (19) |  |
| **Subtrochanteric** | 7,139 (8.2) | 1,679 (7.0) |  |
| **Residential status** |  |  | <.001 |
| **Single person household** | 46,149 (53) | 4,710 (20) |  |
| **Multiple person household** | 29,643 (34) | 4,078 (17) |  |
| **Long-term care resident** | 11,571 (13) | 15,202 (63) |  |
| **Baseline walking ability** | |  | <.001 |
| **Alone outdoors** | 62,028 (71) | 5,522 (23) |  |
| **Only with company outdoors** | 6,136 (7.0) | 3,613 (15) |  |
| **Alone indoors** | 13,609 (16) | 9,989 (42) |  |
| **Only with company indoors** | 3,635 (4.2) | 3,749 (16) |  |
| **Can't walk** | 1,955 (2.2) | 1,117 (4.7) |  |
| **Walking ability after 4 months** | |  | <.001 |
| **Alone outdoors** | 23,058 (46) | 817 (7.7) |  |
| **Only with company outdoors** | 6,001 (12) | 1,111 (10) |  |
| **Alone indoors** | 12,373 (25) | 3,160 (30) |  |
| **Only with company indoors** | 4,639 (9.2) | 2,956 (28) |  |
| **Can't walk** | 4,096 (8.2) | 2,575 (24) |  |
| **(Missing)** | 37,196 | 13,371 |  |
| **Discharged to** |  |  | <.001 |
| **Home** | 33,647 (40) | 1,687 (7.4) |  |
| **Long-term care facility** | 43,047 (51) | 19,926 (87) |  |
| **Another hospital or clinic** | 7,444 (8.8) | 1,161 (5.1) |  |
| **(Missing)** | 3,225 | 1,216 |  |
| **30-day mortality** | 5,220 (6.0) | 3,220 (13) | <.001 |
| **4-month mortality** | 10,836 (12) | 6,444 (27) | <.001 |
| **1-year mortality** | 17,389 (20) | 9,458 (39) | <.001 |

| ^‡^Median (IQR); n (%); ^§^Wilcoxon rank sum test; Pearson's Chi-squared test; ^\|\|^ASA, American Society of Anaesthesiology. |
| --- |

**Supplementary table S2**: Number of dementia cases per diagnosis and average follow-up time post-surgery.

| **Dementia** | **N** | **% of all dementia cases** | **Average follow-up in days (Min-Max)** |
| --- | --- | --- | --- |
| **Late AD** | 1533 | 26% | 738 (15-3403) |
| **Early AD** | 74 | 1% | 710 (12-3063) |
| **Mix of AD and VascD** | 1169 | 20% | 732 (8-3623) |
| **Frontotemporal dementia** | 46 | 1% | 591 (56-1952) |
| **Dementia with Lewy bodies** | 136 | 2% | 665 (36-2267) |
| **Parkinson disease dementia** | 107 | 2% | 821 (17-2414) |
| **No specified dementia** | 1575 | 26% | 732 (11-2867) |
| **Other dementia** | 130 | 2% | 706 (2-2851) |
| **Vascular dementia** | 1174 | 20% | 704 (8-3292) |
| **Total** | 5944 | 100% | 723 (2-3623) |
|  | | |  |

**Supplementary table S3:** Cox regression analysis for dementia specific risk of 1-year mortality following surgically treated hip fracture.

|  | **HR^*^** | **95% CI**† | **p-value** |
| --- | --- | --- | --- |
| **No Dementia** | REF | — |  |
| **Dementia** | 1.05 | 0.95 to 1.15 | 0.08 |
| **Late AD** | 1,03 | 0,93 to 1,14 |  |
| **Early AD** | 1,1 | 0,69 to 1,85 |  |
| **Mix of AD and VascD** | 1,06 | 0,94 to 1,19 |  |
| **VascD** | 1,7 | 0,96 to 1,21 |  |
| **Frontotemporal dementia** | 2,23 | 0,71 to 5,26 |  |
| **Dementia with Lewy bodies** | 0,93 | 0,68 to 1,24 |  |
| **Parkinson disease dementia** | 0,76 | 0,48 to 1,11 |  |
| **No specified dementia** | 1,01 | 0,91 to 1,13 |  |
| **Other dementia** | 1,14 | 0,61 to 1,29 |  |
| * HR, Hazard Ratio; †CI, Confidence Interval**;** ^‡^ASA, American Society of Anaesthesiology | | | |

**Supplemental figure 1:** Dementia diagnosis overlap between individual registries.

**Supplemental figure 2:** Hazard ratios of 1-year mortality per dementia type.
